# Training needs assessment for practicing pediatric critical care nurses in Malawi to inform the development of a specialized master’s education pathway: a cohort study

**DOI:** 10.1186/s12912-021-00772-3

**Published:** 2022-01-04

**Authors:** Kelsey Renning, Brittney van de Water, Shelley Brandstetter, Chisomo Kasitomu, Netsayi Gowero, Miriam Simbota, Maureen Majamanda

**Affiliations:** 1 Pediatric Critical Care Nursing Educator, Seed Global Health, Boston, MA USA; 2 Child Health Lecturer, Kamuzu University of Health Sciences, Blantyre, Malawi; 3 Seed Global Health, Boston, MA USA; 4 Queen Elizabeth Central Hospital, Blantyre, Malawi; 5 Consortium for Advanced Research Training in Africa (CARTA), Nairobi, Kenya

**Keywords:** Critical care nursing, Pediatric intensive care units, Pediatrics, Low resource setting, Nursing education, Global health nursing, Nursing needs assessment

## Abstract

**Background:**

Significant improvements in under-five mortality in Malawi have been demonstrated over the past thirty years; however, Malawian healthcare remains with gaps in availability and access to quality pediatric critical care nursing training and education. To improve expertise of pediatric critical care nurses in Malawi, Kamuzu University of Health Sciences (KUHeS), Queen Elizabeth Central Hospital (QECH), and Mercy James Center (MJC) entered a partnership with Seed Global Health, a US non-governmental organization. A needs assessment was conducted to understand the training needs of nurses currently working in pediatric critical care and in preparation for the development of a specialized Master’s in Child Health pathway in Pediatric Critical Care (PCC) Nursing at KUHeS.

**Methods:**

The needs assessment was completed using a survey questionnaire formatted using an ABCDE (Airway, Breathing, Circulation, Disability, and Exposure) framework. The questionnaire had Likert scale and yes/no questions. Data was manually entered into excel and was analyzed using descriptive statistics.

**Results:**

One hundred and fifty-three nurses at QECH and MJC responded to the survey. Most nurses were between the ages of 25 and 35 years (*N* = 98, 64%), female (*N* = 105, 69%), and held either a Bachelors (*N* = 72, 47%) or diploma (*N* = 70, 46%) in nursing. Nurses had high rates of confidence in certain skills: airway management (*N* = 120, 99%), breathing assessment & management (*N* = 153, 100%). However, nurses demonstrated little to no confidence in areas such as: mechanical ventilation (*N* = 68, 44%), ECG evaluation (*N* = 74, 48%), and arterial blood gas collection & interpretation (*N* = 49, 32%).

**Conclusion:**

It is important to identify priority areas for training and skills development to address in the PCC master’s within the child health pathway at KUHeS. Ideally this partnership will produce practice-ready PCC nurses and will establish a recognized PCC nursing workforce in Malawi.

## Background

In 1990, Malawi had the second highest under-five mortality rate globally with 221 deaths per 1000 live births [1]. Significant improvements in under-five mortality in Malawi have been demonstrated over the past 30 years with recent data showing that the rate has declined to 41.6 deaths per 1000 live births [2]. Malawi is one of the few countries that met the Millennium Development Goal of reducing child mortality by two-thirds by 2015 due to improvements in childbirth and the prevention and treatment of diseases such as pneumonia, diarrhea, malaria, and HIV [3]. Following this accomplishment came the establishment of the agenda for 2030 Sustainable Development Goals (SDGs) which aim at further reducing deaths in the under-five population [4]. The Malawian Ministry of Health (MoH) highlights several strategies and programs aimed at improving health outcomes and access to care at all levels of healthcare from rural to district and central hospitals. These strategies and programs include family planning, access to Skilled Birth Attendants, youth health services, and training health professionals in Helping Babies Breathe (a simulation-based educational program to teach neonatal resuscitation in low-resource settings) [5, 6].

Despite these encouraging statistics, Malawi remains with significant gaps in the availability of quality pediatric critical care and pediatric critical care nursing education and further progress must be made to achieve the health-related SDGs [7, 8]. One explanation for this gap may be the absence of specialized pediatric critical care training for nurses, yet the training needs of PCC nurses in Malawi are not known. It has been shown that level of training, length of nursing experience, and high nurse to patient ratios have been linked to mortality and adverse health outcomes in adult intensive care units, which presumably would be reflected in the pediatric realm [9]. Other factors identified to negatively impact the survival of young children include childbirth outside the hospital, financial constraints, and delayed presentation to the hospital due to lack of community-based healthcare [10–12]. In Malawi, delay in care is often associated with parents providing home remedies to children and only going to the hospital when they perceive an illness to be life-threatening [13]. Consequently, critically ill children often arrive too late to the hospital for life-saving treatment, resulting in immediate (within 4 h) or early (within 48 h) death [14].

To improve child mortality rates and better prepare nurses in Malawi, the researchers sought to perform a training needs assessment among practicing nurses in the pediatric units for critically ill children at Queen Elizabeth Central Hospital (QECH) and Mercy James Centre for Paediatric Surgery and Intensive Care (MJC) in Blantyre, Malawi. The findings from this assessment will help inform the development of a specialized Pediatric Critical Care (PCC) track within the Master’s in Child Health Nursing Program at the Kamuzu University of Health Sciences (KUHeS, formerly Kamuzu College of Nursing/KCN).

### Partnership plan development

KUHeS and QECH have been imparting clinical skills to nursing students in partnership since the opening of the nursing college in 1965. In the past decade, new initiatives have evolved at QECH with support from KUHeS child health members to address the burden of childhood illness, such as emergency care, High Dependency Unit (HDU) care, and nutritional rehabilitation. In 2017, with support from Raising Malawi and other stakeholders, QECH built the first pediatric critical care center in Malawi – MJC – comprised of a 6-bed pediatric intensive care unit (PICU), three operating theatres, HDUs, and a surgical ward [15].

To improve nurses’ expertise of pediatric critical care, KUHeS, QECH, and MJC entered a partnership with Seed Global Health (SGH), an American non-governmental organization [16]. From 2013 to 2018, KUHeS and SGH were connected through the Peace Corps’ Global Health Service Partnership (GHSP) program. The goal of the current partnership is to create a pathway to a specialized PCC master’s that would produce a formally recognized pediatric critical care nursing workforce by 2024.

## Methods

### Design

A search for validated or published needs assessment tools for pediatric critical care nursing in low-resource settings returned no relevant results. Broadening the search to include “needs assessment,” “pediatric,” and “low-income” keywords delivered 204 results, 17 of which were reviewed for relevance and were not applicable. Further searches for training needs assessments for pediatric critical care nursing in any setting returned only one validated tool, which lacked validation in other settings and was deemed irrelevant for this project [17]. Therefore, the researchers developed a tool specifically for pediatric critical care nursing skills in Malawi.

A quantitative approach in the form of a questionnaire using an ABCDE framework with Likert-scale and yes/no questions was created. Additional questions asked about experience, current employment location, and future career aspirations to help illustrate any difference in learning needs. The tool underwent face validity testing with senior pediatric nursing faculty and was thought to truly measure skill competence (See Appendix A: Needs Assessment Questionnaire) [18].

No patients or members of the public were involved in the design, or conduct, or reporting, or dissemination plans of our research.

### ABCDE framework

Researchers developed a thorough, yet succinct, questionnaire covering specific components of critical care nursing using an “ABCDE” framework. This “ABCDE” format is used in nursing assessment and documentation and includes assessment of Airway, Breathing, Circulation, Disability, and Exposure (ABCDE) domains [19]. Questions were based on expected PICU competencies covered during new nurse training at MJC and were focused on assessing knowledge and confidence in executing nursing skills [20]. To cover topics that are important in pediatric critical care nursing, yet not part of this ABCDE framework, thematic areas of infection prevention and family education were added. The questionnaire also included demographic, educational, and experiential items.

There was a total of three questions related to Airway, seven related to Breathing, fifteen related to Circulation, six related to Disability, and four related to Exposure. There were two questions related to Emergency Preparedness, three questions related to Infection Prevention, and one question related to Family Education. Each question had a Likert scale of 1–4 with 1 being “Not confident,” 2 being “Somewhat confident” and 3 being “Confident” in that skill. A fourth option was “Unsure/I don’t know.”

### Setting and population

The training needs assessment questionnaire was distributed to 165 practicing nurses working in nine pediatric departments at QECH and MJC. The nine departments include: Pediatric Accident and Emergency (*N* = 20), Pediatric Special Care Ward HDU (*N* = 18), Pediatric Nursery HDU (*N* = 19), Nutritional Rehabilitation Unit HDU (*N* = 8), Main ICU (*N* = 19), Neurology HDU (*N* = 13), Chatinkha Nursery HDU (*N* = 24), MJC HDU (*N* = 18), and MJC PICU (*N* = 26).

### Data collection and analysis

The electronic questionnaire was distributed to nurses via email using Google Forms. Data were collected over 2 weeks and were entered into an excel spreadsheet for descriptive analysis.

## Results

The questionnaire was completed by 153 practicing nurses (153/165, 92.7% response rate). 77% of nurses were under the age of 35 (*N* = 118), 69% were female (*N* = 105), 75% were permanent employees (*N* = 115), 93% worked in pediatrics (142), 46% held a diploma (*N* = 70), and 47% held a bachelor’s degree (*N* = 72). The setting/specialty question was in multiple choice format; thus, the percentage of respondents does not add up to 100% reflecting nurses who work in multiple wards (Table 1).

**Table 1:**
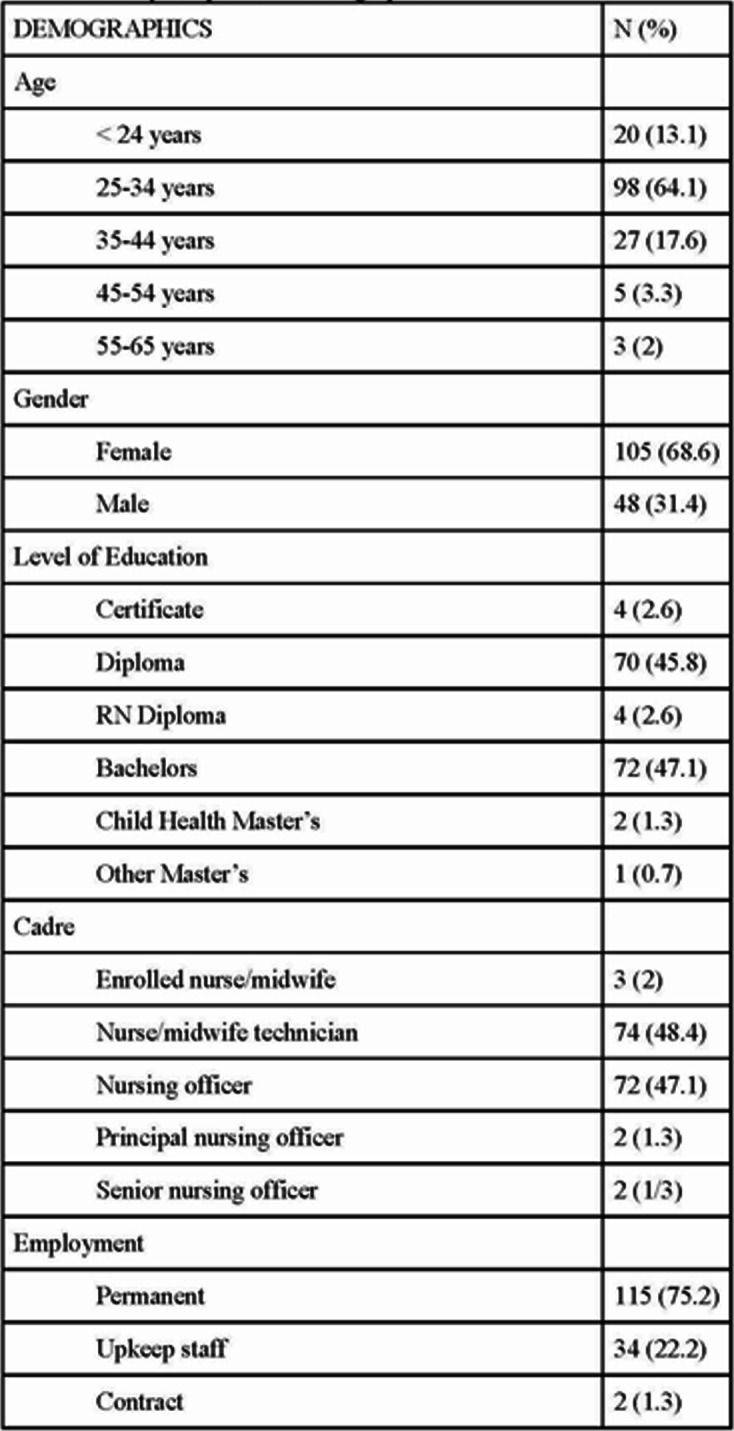
Survey respondent demographics

### Airway

Nurses were confident (*N* = 90, 58.8%) or somewhat confident (*N* = 60, 39.2%) in airway assessment and management. Specifically, nurses felt somewhat confident (*N* = 55, 35.9%) in endotracheal securing, monitoring, and management. However, 35 nurses (22.9%) were not confident in this skill. Over three-quarters of nurses felt confident in safe airway suctioning and nebulization (*N* = 118, 77.1%).

### Breathing

Nurses were confident (*N* = 136, 88.9%) or somewhat confident (*N* = 17, 11.1%) in breathing assessment and management. Nurses were confident (*N* = 62, 40.5%) or somewhat confident (*N* = 72, 47.1%) in performing respiratory therapies. Many (*N* = 68, 44.4%) were somewhat confident in arterial blood gas collection, however there were nurses who were either confident (*N* = 36, 23.5%) or not confident (*N* = 32, 20.9%). Results varied regarding use of mechanical ventilation equipment (modes, settings, troubleshooting) with an almost even split between nurses feeling confident (*N* = 37, 24.2%), somewhat confident (*N* = 50, 32.7%), and not confident (*N* = 45, 30.1%). Nurses were similarly split when it came to care of a mechanically ventilated patient (monitoring, interpretation, care before and after extubating) and felt confident (*N* = 48, 31.4%), somewhat confident (*N* = 37, 24.2%), and not confident (*N* = 46, 30.1%). Most nurses felt confident (*N* = 93, 60.8%) or somewhat confident (*N* = 37, 24.2%) in use of bubble continuous positive airway pressure (bCPAP) equipment, patient monitoring, and troubleshooting.

### Circulation

Most nurses (*N* = 151, 98.7%) were confident or somewhat confident in assessment of circulation. Nurses were somewhat confident (*N* = 53, 34.6%) or not confident (*N* = 46, 30.1%) in electrocardiogram (ECG) evaluation, however some nurses did feel confident (*N* = 26, 17%) or were unsure (*N* = 28, 18.3%) of this skill. Less than half of nurses (*N* = 69, 45.1%) felt somewhat confident in performing a cardiac assessment, while other nurses felt confident (*N* = 36, 23.5%) or not confident (*N* = 37, 24.2%). Most nurses felt confident (*N* = 52, 34%) or somewhat confident (*N* = 73, 47.7%) in preparing and administering vasoactive medications (i.e., adrenaline), while some still felt not confident (*N* = 22, 14.4%). Nurses were confident (*N* = 58, 37.9%) or somewhat confident (*N* = 47, 30.7%) in arterial line management (sample collection, dressing change, infection prevention, troubleshooting), while fewer were not confident (*N* = 28, 18.3%) or unsure (*N* = 23, 15%). Regarding central line management (sample collection, dressing change, infection prevention, troubleshooting), most nurses felt confident (*N* = 62, 40.5%) or somewhat confident (*N* = 47, 30.7%), however many still felt not confident (*N* = 21, 13.7%) or unsure (*N* = 23, 15%). Nurses (*N* = 149, 97.3%) felt confident or somewhat confident in peripheral IV management. Most nurses were somewhat confident (*N* = 51, 33.3%), not confident (*N* = 64, 41.8%), or unsure (*N* = 23, 15%) about intraosseous cannula management, while only few nurses (*N* = 15, 9.8%) were confident in this skill. Nurses felt somewhat confident (*N* = 61, 39.9%) in chest tube management. Others were split between confident (*N* = 35, 22.9%), not confident (*N* = 38, 24.8%), or unsure (*N* = 19, 12.5%). Nurses overall were confident or somewhat confident in managing dehydration (*N* = 151, 98.7%), fluid overload (*N* = 150, 98.1%), urinary catheters (*N* = 148, 96.7%), and assessing urine output (*N* = 149, 97.4%).

### Disability

Nurses were confident or somewhat confident in neurological status assessment (*N* = 145, 94.8%), blood glucose monitoring (*N* = 150, 98.1%), nasogastric tube management (*N* = 153, 100%), total parenteral nutrition management (*N* = 130, 84.9%), pain assessment (*N* = 149, 97.4%). While most nurses felt confident (*N* = 48, 31.4%) or somewhat confident (*N* = 70, 45.8%) with sedation management, some nurses did not feel confident (*N* = 26, 17%).

### Exposure

Nurses were confident or somewhat confident in management of hypothermia (*N* = 103, 100%) and hyperthermia (*N* = 153, 100%), skin integrity (*N* = 152, 99.4%), and head-to-toe assessment (*N* = 148, 96.8%).

### Emergency preparedness, infection prevention, and family education

Nurses were confident or somewhat confident in accessing emergency supplies (*N* = 146, 95.5%), recognizing a deteriorating child (*N* = 150, 98.1%), hand hygiene (*N* = 152, 99.3%), donning and doffing protective wear (*N* = 152, 99.3%), decontamination of equipment (*N* = 150, 98%), and providing family education (*N* = 150, 98.7%) (see Table 2).

**Table 2:**
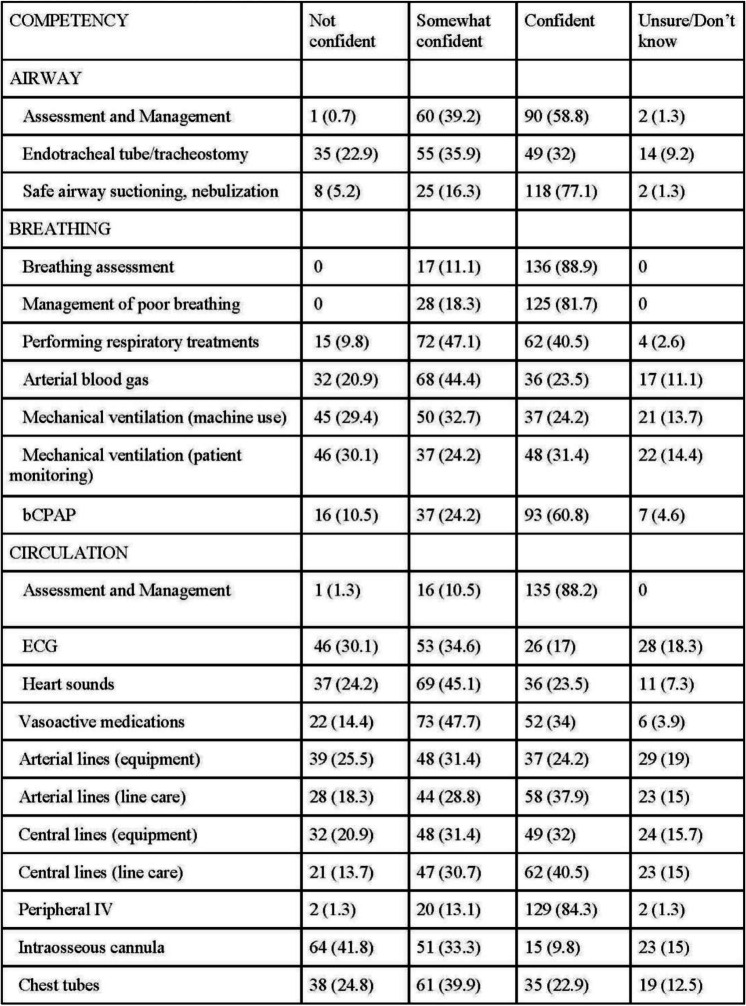

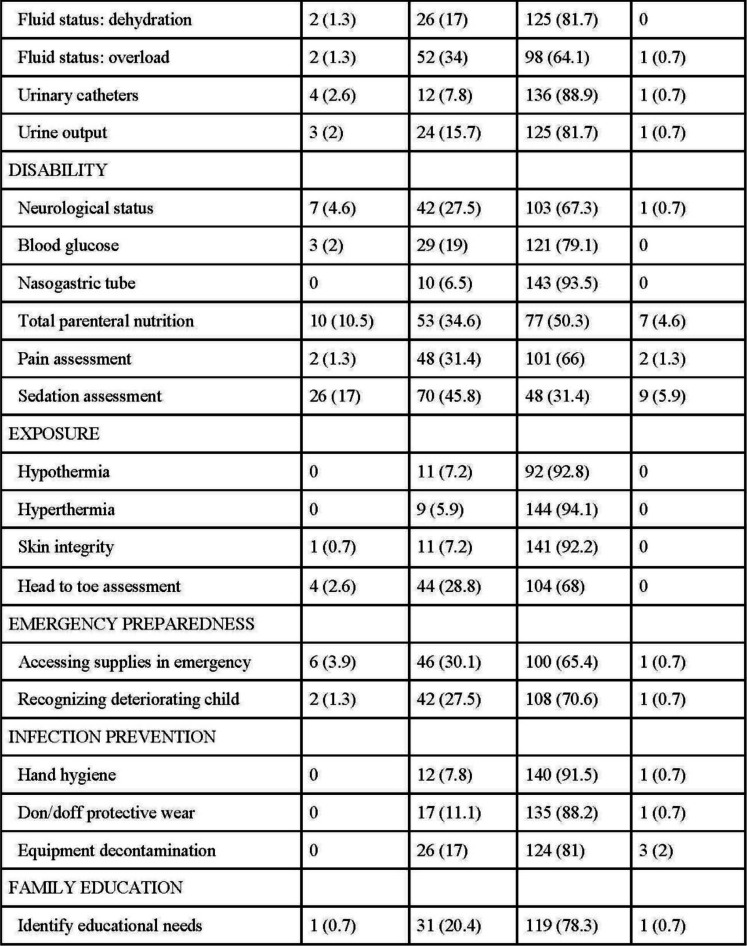
ABCDE competency responses

### Work environment

Nurses were asked questions about the number of patients they work with and the equipment they use. Many nurses cared for either 1–3 patients at a time (*N* = 56, 36.6%) or greater than 17 patients (*N* = 38, 24.8%). Most nurses indicated that they do not have adequate equipment (bag valve masks, oxygen, bCPAP machines) (*N* = 124, 81.1%) or supplies (gloves, aprons, masks, etc.) (*N* = 113, 73.8%) required to perform essential job duties. However, most nurses indicated that the equipment they use does function properly (*N* = 140, 91.5%). Almost all nurses indicated that they are comfortable asking a colleague, supervisor (*N* = 152, 99.3%), or medical doctor (*N* = 149, 97.4%) questions about patient care (see Table 3).

**Table 3:**
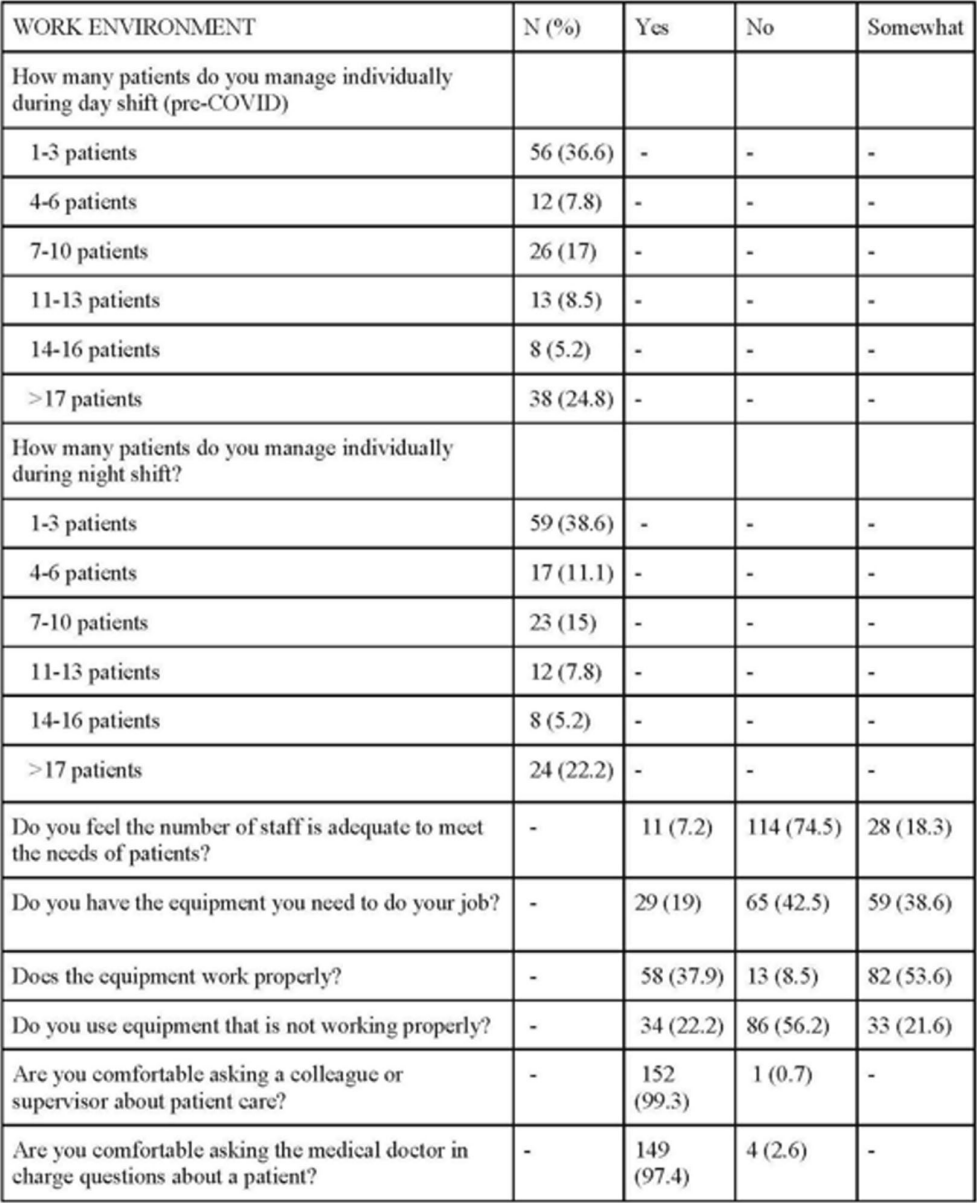
Work environment

Nurses were asked about their desire to stay in their current ward, desire to pursue a PCC master’s, learning style, and past training. 76.5% of nurses (*N* = 117) indicated a desire to remain in their current unit for 5 years or more, 88.9% (*N* = 136) indicated a desire to pursue a PCC master’s, and 80.4% (*N* = 123) prefer hands-on or simulation learning style. There were low rates of pediatric critical care training despite working in pediatric critical care areas. For example, only 65% of nurses had training in Emergency Triage, Assessment & Treatment (ETAT), and 52% had training in bCPAP. This question was written in multiple choice format, so some nurses may have indicated both ETAT and bCPAP training (see Table 1).

## Discussion

Needs assessments alone are not enough to impact patient health outcomes, so this study is the beginning of a larger education and practice change [21]. Nurses with master’s degrees have advanced nursing roles which enable them to provide clinical leadership, positively impact clinical practice, and improve the provision of care [22]. The findings indicate that only two participants were trained at the master’s level, and these are principal and senior nursing officers. As such, their primary focus is on managerial and administrative duties rather than to provide bedside nursing care. This calls for more nurses to be trained at the master’s level so that they can provide leadership in quality care of critically ill children.

Overall, there were high rates of critical care confidence among nurses. For example, nearly all nurses felt confident or somewhat confident in assessment and management of skills across all ABCDE domains. A study in Uganda and Zambia showed that healthcare providers’ confidence increased with exposure to a variety of skills, which will be important to consider when designing the content of a PCC master’s program [23].

Nurses rated high confidence particularly in task-oriented or scale-dependent skills, including the Glasgow or Blantyre Coma Scale, blood glucose monitoring, nasogastric tube management, pain assessment, temperature monitoring, skin assessment, and infection prevention. Further investigation may be revealing as to whether nurses know how to interpret the data they collect, what intervention is indicated based on the findings, and when to reassess the patient, which would better demonstrate critical thinking skills and a higher level of nursing competency [24].

Although nearly 97% of nurses indicated “confident” or “somewhat confident” to performing head-to-toe assessments and focused cardiac and pulmonary assessments, nurses indicated lower levels of confidence in listening to heart sounds specifically. This may indicate a gap in nursing knowledge, incomplete or rushed assessments, or a lack of understanding of how to interpret assessment findings. Comprehensive head-to-toe and focused assessments are more meaningful if nurses can interpret the findings, anticipate interventions, make recommendations, and monitor a patient’s response to the intervention because these actions help in identifying a patient whose condition is deteriorating. It will be important to identify the underlying cause of nurses’ lack of confidence as a matter of patient safety [25, 26].

High patient volumes could affect the nurses’ ability to confidently perform assessments. Half the nurses care for 1–3 or 4–6 patients on a shift, which is comparable to some high-income settings such as California, USA, where state law requires a ratio of 1:2 for critical care wards and up to 1:6 in medical/surgical wards [27]. However, one third of nurses care for > 17 patients and 75–80% of nurses feel their ward is understaffed and underequipped. While almost 100% of nurses feel comfortable asking a colleague, supervisor, or medical doctor a question about their patient’s care, this still has great implications for the nurse’s ability to dedicate quality time to individual patients, provide thorough education, and quickly recognize clinical deterioration [28]. High patient workload in pediatric critical care settings is associated with long hospital stays and poor health outcomes for patients, and for nurses, increased medical errors and burn out [28–30].

Findings highlighted other areas where most of the nurses were less confident. Half the nurses were not confident or somewhat confident in managing endotracheal tubes or tracheostomies, and a little over half of nurses were not confident or somewhat confident in managing mechanical ventilation. This was not a surprising finding as the only ventilators are located at PICU at MJC and in the main ICU at QECH, therefore very few nurses provide care for ventilated patients. Additionally, intubation is not a skill that nurses are permitted to perform or gain competency in and so less familiarity with the procedure may impact confidence in managing related equipment such as endotracheal tubes and ventilators [23].

Infrequent exposure may have also impacted nurses’ low confidence in ECG interpretation despite nearly 90% confidence with cardiac assessments, as there is no dedicated pediatric cardiac unit and continuous ECG monitoring is not available on most of the pediatric units. Children with cardiac problems receive the same monitoring that other children receive: oxygen saturation, pulse rate, and temperature. Blood pressure and respirations are not routinely monitored in wards with high patient volume. Conversely, in the PICU at MJC, continuous monitoring is available, and the nurse-to-patient ratio is generally 1:1 or 1:2. Despite low confidence in ECG monitoring, most nurses (~ 80%) rated confident or somewhat confident their knowledge and use of vasoactive medications like adrenaline (used in cardiopulmonary resuscitation). When considering that many pediatric patients are very sick by the time they reach the hospital, the nurse’s confidence in using such a medication could be reflected simply by its frequency of use.

Considering effective learning styles for nurses, simulation has been shown to greatly impact nursing students’ self-efficacy and performance of nursing skills [31]. When asked about learning preferences, 80% preferred hands-on simulation and nearly half of nurses were interested in e-learning technologies, while 68% reported bedside teaching and classroom learning were preferred. Furthermore, combining learning styles has been shown to improve nursing students’ motivation, satisfaction, and subject-specific knowledge when compared to online-only learning [32]. Providing varied and accessible learning modalities through simulation, flipped classroom, and online curriculum will be important for ensuring success among nursing students as the PCC pathway is being created.

Research suggests that nurse retention in Malawi is negatively impacted by poor compensation, poor working conditions, high patient ratios, unsupportive managerial relationships, lack of career advancement, and lack of performance evaluation [33]. It has conversely been shown that nurse retention in rural settings is positively impacted by a clear career pathway, mentorship, and financial incentives [34]. In Malawi specifically there are strong religious and patriotic factors that increase retention [33]. Supportive work environments increase nurses’ job satisfaction, but have the added benefit of positively affecting patient and family satisfaction [35, 36]. Encouragingly, 76.5% of nurses had a desire to stay in their current ward for 5 years or more, and 89% of nurses were interested in pursuing a PCC master’s degree (if funding were attainable). These findings may represent protective factors positively impacting nurse retention rates and a desire for specialization. Creating a PCC master’s pathway will give nurses an opportunity to acquire specialized skills which would encourage the MoH and hospital leadership to allow nurses to work within their area of expertise, improving nurses’ job satisfaction and ultimately patient care.

### Limitations

This study is not without limitations. While English is the official language of Malawi, it is commonly nurses’ second language which could contribute to some measurement error. This study was conducted at one tertiary facility and as such findings cannot be generalized. The nurses work in a wide range of wards, and due to the inability to complete a comparative analysis, we are unable to determine the differences in responses by nurses from ward to ward. Although the questionnaire was considered confidential, data was deidentified for analysis, and answers were not shared with clinical department heads (eliminating the risk of repercussions for low-confidence responses), nurses may have reported over-confidence in their skills due to an inherent desire to prove clinical competence and adhere to unit standards. Additionally, we must recognize that self-rated confidence may not transfer to validated competence and improved patient outcomes.

## Conclusion

Among 153 practicing nurses we found that nurses indicated a high level of confidence across all ABCDE domains and in task or scale-dependent skills like NG tube management, pain assessment, and coma scores. We were able to better understand nurses’ training needs related to pediatric critical care; most gaps were in mechanical ventilation, ECG evaluation and cardiac assessment, intraosseous cannulation, and chest tube management. Confidence in some skills was variable, possibly dependent on frequency of exposure to that skill. Findings demonstrated a possible knowledge gap between performing assessments and interpreting assessment findings. High patient volume could be impacting confidence in assessments. Concerns regarding poor staffing and inadequate equipment were also highlighted.

The nurses’ responses have illuminated themes that occur commonly in low-resource settings, and therefore these findings could be generalized to benefit similar low-resource settings around the world. In Malawi, this study will contribute to the development of a PCC master’s pathway. It will be important to involve the Ministry of Education and MoH in the planning of this program to best employ nurses and to better serve the children in need of high acuity nursing care.

## Data Availability

No additional data. The data that support the findings of this study are available from the corresponding author, KR, upon reasonable request.

## Appendix A: Needs Assessment Questionnaire

**TRAINING NEEDS ASSESSMENT SURVEY FOR NURSES.**

SECTION A: Background Questions for Practicing Nurses

1. Age (years)
  - 24 and below
  - 25–34
  - 35–44
  - 45–54
  - 55–65
  - Above 65

2. Sex:
  - Male
  - Female

3. What is your highest professional education?
  - Certificate
  - Diploma
  - RN Diploma
  - BSc
  - Child Health MSc
  - MSc (Other)
  - PhD

4. Cadre
  - Enrolled nurse/Midwife
  - Nurse/Midwife technician
  - Nursing Officer
  - Senior Nursing Officer
  - Principal Nursing officer
  - Chief Nursing officer

5. Employment status
  - Permanent
  - Voluntary
  - Locum
  - Intern
  - Upkeep
  - Contract

6. How many years have you worked as a nurse/midwife?
  - Less than 1 year
  - 1–4
  - 5–9
  - 10–14
  - 15–19
  - 20 and above

7. What areas of nursing/midwifery have you worked in? Tick all that apply.
  - Paediatrics
  - Midwifery
  - Community/ Occupational
  - Adult Wards
  - Psychiatric

8. Which ward/unit are you currently working? Tick all that apply.
  - Paediatric A&E
  - Paediatric Special Care Ward HDU
  - Paediatric Nursery HDU
  - Nutritional Rehabilitation Unit (NRU) HDU
  - Main ICU
  - Neurology HDU
  - Chatinkha Nursery HDU
  - Mercy James Centre – HDU
  - Recovery
  - Burns HDU
  - Mercy James Centre ICU

9. What other paediatric critical care related training have you received? Tick all that apply.
  - Advanced Burn Life Support (ABLS)
  - Advanced Paediatric Life Support (APLS)
  - Emergency Triage Assessment and Treatment (ETAT)
  - Nursing Education Partnership Initiative Preceptor Training
  - Bubble Continuous Positive Airway Pressure Training (BCPAP)
  - Care of the Infant and Newborn Training (COIN)
  - Basic Life Support (BLS)
  - Palliative Care
  - Other

10. If it were up to you, for how long would you want to stay in your current ward/unit:
  - I want to leave soon
  - 1 year or less
  - 2–4 years
  - more than 5 years

11. If funding allowed, would you be interested to pursue a MSc in Child Health Critical Care Nursing?
  - Yes
  - Maybe
  - No

12. How do you like to learn new information? Tick all that apply.
  - Reading
  - Classroom/Video
  - Hands-On Practice/Simulation
  - E-learning
  - Bedside teaching/learning

SECTION B: Current Level of Competencies (ABCDE Knowledge & Skills) in PCC Nursing.

Please answer truthfully and to the best of your ability. Not all of the listed skills may be relevant to your specific work area, but please select an answer for each item.

AIRWAY

13. Assessment and management of airway (positioning, log rolling, jaw thrust, chin lift, and Heimlich manoeuvres)
  - Unsure / Don’t Know
  - Not confident: Could not safely do on your own
  - Somewhat confident: Would perform with help or under supervision
  - Confident: Could teach someone else

14. Endotracheal Tube / Tracheostomy: Securing, monitoring and management (Patency, correct length, cuff leakage)
  - Unsure / Don’t Know
  - Not confident: Could not safely do on your own
  - Somewhat confident: Would perform with help or under supervision
  - Confident: Could teach someone else

15. Safe Airway Suctioning and nebulization (naso/oropharyngeal, endotracheal tube, tracheostomy)
  - Unsure / Don’t Know
  - Not confident: Could not safely do on your own
  - Somewhat confident: Would perform with help or under supervision
  - Confident: Could teach someone else

BREATHING

16. Breathing assessment (Resp rate, oxygen sats, breathing problems and identification of abnormal sounds)
  - Unsure / Don’t Know
  - Not confident: Could not safely do on your own
  - Somewhat confident: Would perform with help or under supervision (regardless of actual help available)
  - Confident: Could teach someone else

17. Management of poor breathing mechanisms (oxygen therapy, bag and mask ventilation)
  - Unsure / Don’t Know
  - Not confident: Could not safely do on your own
  - Somewhat confident: Would perform with help or under supervision (regardless of actual help available)
  - Confident: Could teach someone else

18. How to perform Respiratory Treatments or Therapies (i.e., metered dose inhaler, chest physiotherapy, nebulizing medication)
  - Unsure / Don’t Know
  - Not confident: Could not safely do on your own
  - Somewhat confident: Would perform with help or under supervision (regardless of actual help available)
  - Confident: Could teach someone else

19. Arterial blood Gas: collecting blood sample, running a test and interpretation
  - Unsure / Don’t Know
  - Not confident: Could not safely do on your own
  - Somewhat confident: Would perform with help or under supervision (regardless of actual help available)
  - Confident: Could teach someone else

20. Mechanical Ventilation: Different modes, setting, responding, and addressing alarms on machine and troubleshooting.
  - Unsure / Don’t Know
  - Not confident: Could not safely do on your own
  - Somewhat confident: Would perform with help or under supervision (regardless of actual help available)
  - Confident: Could teach someone else

21. Mechanical Ventilation: connecting to a patient, monitoring, interpretation, management before and after extubating.
  - Unsure / Don’t Know
  - Not confident: Could not safely do on your own
  - Somewhat confident: Would perform with help or under supervision (regardless of actual help available)
  - Confident: Could teach someone else

22. bCPAP: Indications for use, initiation, machine set-up including choice of nasal prongs, escalating and weaning treatment, proper documentation and troubleshooting.
  - Unsure / Don’t Know
  - Not confident: Could not safely do on your own
  - Somewhat confident: Would perform with help or under supervision (regardless of actual help available)
  - Confident: Could teach someone else

CIRCULATION

23. Assessment, interpretation, management, and documentation of impaired circulation (warmth of extremities, capillary refill time, pulse, blood pressure)
  - Unsure / Don’t Know
  - Not confident: Could not safely do on your own
  - Somewhat confident: Would perform with help or under supervision (regardless of actual help available)
  - Confident: Could teach someone else

24. Evaluating Electrocardiogram (ECG) for Rate and Rhythms (Normal, Arrhythmias)
  - Unsure / Don’t Know
  - Not confident: Could not safely do on your own
  - Somewhat confident: Would perform with help or under supervision (regardless of actual help available)
  - Confident: Could teach someone else

25. How to listen to heart sounds, interpretation, and documentation.
  - Unsure / Don’t Know
  - Not confident: Could not safely do on your own
  - Somewhat confident: Would perform with help or under supervision (regardless of actual help available)
  - Confident: Could teach someone else

26. Vasoactive Medications: preparation, administration, Monitoring (e.g., Adrenaline, Dopamine),
  - Unsure / Don’t Know
  - Not confident: Could not safely do on your own
  - Somewhat confident: Would perform with help or under supervision (regardless of actual help available)
  - Confident: Could teach someone else

27. Arterial lines: knowledge of equipment, assessment, and interpretation
  - Unsure / Don’t Know
  - Not confident: Could not safely do on your own
  - Somewhat confident: Would perform with help or under supervision (regardless of actual help available)
  - Confident: Could teach someone else

28. Arterial lines: Management (sample collection, changing dressing, infection prevention, oedema, extravasation, safe removing, troubleshooting)
  - Unsure / Don’t Know
  - Not confident: Could not safely do on your own
  - Somewhat confident: Would perform with help or under supervision (regardless of actual help available)
  - Confident: Could teach someone else

29. Central lines: knowledge of equipment, assessment, and interpretation
  - Unsure / Don’t Know
  - Not confident: Could not safely do on your own
  - Somewhat confident: Would perform with help or under supervision (regardless of actual help available)
  - Confident: Could teach someone else

30. Central lines: Management (sample collection, changing dressing, infection prevention, oedema, extravasation, safe removing, troubleshooting)
  - Unsure / Don’t Know
  - Not confident: Could not safely do on your own
  - Somewhat confident: Would perform with help or under supervision (regardless of actual help available)
  - Confident: Could teach someone else

31. Peripheral IV cannula: Insertion, assessment, monitoring and management (tissuing/ extravasation, dislodgement or leaking, infection prevention, removal).
  - Unsure / Don’t Know
  - Not confident: Could not safely do on your own
  - Somewhat confident: Would perform with help or under supervision (regardless of actual help available)
  - Confident: Could teach someone else

32. Intraosseous cannula: Insertion, assessment, monitoring and management (tissuing/ extravasation, dislodgement or leaking, infection prevention, removal).
  - Unsure / Don’t Know
  - Not confident: Could not safely do on your own
  - Somewhat confident: Would perform with help or under supervision (regardless of actual help available)
  - Confident: Could teach someone else

33. Chest Tubes: Indications/ Use, monitoring, interpretation, management. (accidental removal, securing and troubleshooting)
  - Unsure / Don’t Know
  - Not confident: Could not safely do on your own
  - Somewhat confident: Would perform with help or under supervision (regardless of actual help available)
  - Confident: Could teach someone else

34. Fluid Status: assessment and treatment of dehydration
  - Unsure / Don’t Know
  - Not confident: Could not safely do on your own
  - Somewhat confident: Would perform with help or under supervision (regardless of actual help available)
  - Confident: Could teach someone else

35. Fluid Status: assessment and treatment of fluid overload
  - Unsure / Don’t Know
  - Not confident: Could not safely do on your own
  - Somewhat confident: Would perform with help or under supervision (regardless of actual help available)
  - Confident: Could teach someone else

36. Urinary catheters: Insertion, management, and removal.
  - Unsure / Don’t Know
  - Not confident: Could not safely do on your own
  - Somewhat confident: Would perform with help or under supervision (regardless of actual help available)
  - Confident: Could teach someone else

37. Urine Output: Assessment, interpretation, management
  - Unsure / Don’t Know
  - Not confident: Could not safely do on your own
  - Somewhat confident: Would perform with help or under supervision (regardless of actual help available)
  - Confident: Could teach someone else

DISABILITY

38. Neurological status: Assessment, monitoring, interpretation, and management (AVPU, Blantyre Coma Score, Glasgow Coma Score, reflexes, pupil reactions, warning signs).
  - Unsure / Don’t Know
  - Not confident: Could not safely do on your own
  - Somewhat confident: Would perform with help or under supervision (regardless of actual help available)
  - Confident: Could teach someone else

39. Blood Glucose: assessment Monitoring, interpretation, and management (hypo/hyper glycaemia, sliding scale)
  - Unsure / Don’t Know
  - Not confident: Could not safely do on your own
  - Somewhat confident: Would perform with help or under supervision (regardless of actual help available)
  - Confident: Could teach someone else

40. Nasogastric Tube Insertion and Assessment for proper placement and management
  - Unsure / Don’t Know
  - Not confident: Could not safely do on your own
  - Somewhat confident: Would perform with help or under supervision (regardless of actual help available)
  - Confident: Could teach someone else

41. Total Parenteral Nutrition: preparation, administration, monitoring and management
  - Unsure / Don’t Know
  - Not confident: Could not safely do on your own
  - Somewhat confident: Would perform with help or under supervision (regardless of actual help available)
  - Confident: Could teach someone else

42. Pain assessment and interpretation, monitoring and management
  - Unsure / Don’t Know
  - Not confident: Could not safely do on your own
  - Somewhat confident: Would perform with help or under supervision (regardless of actual help available)
  - Confident: Could teach someone else

43. Sedation assessment, interpretation, monitoring and management
  - Unsure / Don’t Know
  - Not confident: Could not safely do on your own
  - Somewhat confident: Would perform with help or under supervision (regardless of actual help available)
  - Confident: Could teach someone else

EXPOSURE

44. Assessment and management of hypothermia
  - Unsure / Don’t Know
  - Not confident: Could not safely do on your own
  - Somewhat confident: Would perform with help or under supervision (regardless of actual help available)
  - Confident: Could teach someone else

45. Assessment and management of hyperthermia
  - Unsure / Don’t Know
  - Not confident: Could not safely do on your own
  - Somewhat confident: Would perform with help or under supervision (regardless of actual help available)
  - Confident: Could teach someone else

46. Skin integrity: Patient turning and positioning, mouth care, pressure area care
  - Unsure / Don’t Know
  - Not confident: Could not safely do on your own
  - Somewhat confident: Would perform with help or under supervision (regardless of actual help available)
  - Confident: Could teach someone else

47. Head to Toe assessment and management (Injury)
  - Unsure / Don’t Know
  - Not confident: Could not safely do on your own
  - Somewhat confident: Would perform with help or under supervision (regardless of actual help available)
  - Confident: Could teach someone else

EMERGENCY PREPAREDNESS

48. How confident are you to access supplies in your unit in an emergency situation (intubation equipment, ETTs, oral airways, ambu bags, etc.)?
  - Unsure / Don’t Know
  - Not confident: Could not safely do on your own
  - Somewhat confident: Would perform with help or under supervision (regardless of actual help available)
  - Confident: Could teach someone else

49. How confident are you to recognize and resuscitate a deteriorating child? (Airway, Breathing, Circulation, Coma, Convulsions, Dehydration)
  - Unsure / Don’t Know
  - Not confident: Could not safely do on your own
  - Somewhat confident: Would perform with help or under supervision (regardless of actual help available)
  - Confident: Could teach someone else

INFECTION PREVENTION

50. Knowledge of 5 Moments of hand hygiene and how to perform proper hand hygiene according to WHO guideline.
  - Unsure / Don’t Know
  - Not confident: Could not safely do on your own
  - Somewhat confident: Would perform with help or under supervision (regardless of actual help available)
  - Confident: Could teach someone else

51. How and when to don and doff protective wear (gloves, mask, Apron)
  - Unsure / Don’t Know
  - Not confident: Could not safely do on your own
  - Somewhat confident: Would perform with help or under supervision (regardless of actual help available)
  - Confident: Could teach someone else

52. Cleaning and decontamination of equipment according to national IP guidelines
  - Unsure / Don’t Know
  - Not confident: Could not safely do on your own
  - Somewhat confident: Would perform with help or under supervision (regardless of actual help available)
  - Confident: Could teach someone else

FAMILY EDUCATION

53. Assess, identify, and meet the educational needs of the family/caregiver and counsel them accordingly
  - Unsure / Don’t Know
  - Not confident: (Could not safely do on your own)
  - Somewhat confident: (Would perform with help or under supervision)
  - Confident:(Could teach someone else)

SECTION C: Work environment

54. How many patients do you manage individually during day shift (before COVID-19 pandemic)?
  - 1–3
  - 4–6
  - 7–10
  - 11–13
  - 14–16
  - over 17

55. How many patients do you manage individually during night shift (before COVID-19 pandemic)?
  - 1–3
  - 4–6
  - 7–10
  - 11–13
  - 14–16
  - over 17

56. Do you feel the number of staff (including support staff such as hospital cleaners, patient attendants, etc.) is adequate to meet the needs of the patient on the average shift (before COVID-19 pandemic)?
  - Yes
  - Somewhat
  - No

57. Do you have adequate equipment you need to do your work?
  - Yes
  - Somewhat
  - No

58. If “No” or “Sometimes” to Question 57, which equipment is most often or missing? Write your response on the space provided

59. Does the equipment work properly?
  - Yes
  - Somewhat
  - No

60. Do you have to use equipment that is not properly working e.g., ambu bags, oxygen concentrators and bCPAP machines?
  - Yes
  - Somewhat
  - No

61. Do you feel you have enough supplies to do your work, e.g., aprons, gloves, masks, patient medications, lab supplies, etc.?
  - Yes
  - Somewhat
  - No

62. If the answer to question 61 was “No,” which supplies are missing?

63. Are you comfortable asking a colleague or supervisor questions about patient care?
  - Yes
  - Somewhat
  - No

64. Are you comfortable to ask the medical doctor in charge when you have questions about a patient?
  - Yes
  - Somewhat
  - No

## Abbreviations

ABCDE: Airway, breathing, circulation, disability, exposure
bCPAP: Bubble continuous positive airway pressure
ECG: Electrocardiogram
ETAT: Emergency triage, assessment & training
GHSP: Global health service partnership
KCN: Kamuzu College of Nursing
KUHeS: Kamuzu University of Health Sciences
HDU: High dependency unit
MoH: Ministry of health
MJC: Mercy James Centre
PCC: Pediatric critical care
PICU: Pediatric intensive care unit
QECH: Queen Elizabeth Central Hospital
SDGs: Sustainable development goals
SGH: Seed global health
